# Electric-Field Molecular Fingerprinting to Probe Cancer

**DOI:** 10.1021/acscentsci.4c02164

**Published:** 2025-04-09

**Authors:** Kosmas V. Kepesidis, Philip Jacob, Wolfgang Schweinberger, Marinus Huber, Nico Feiler, Frank Fleischmann, Michael Trubetskov, Liudmila Voronina, Jacqueline Aschauer, Tarek Eissa, Lea Gigou, Patrik Karandušovsky, Ioachim Pupeza, Alexander Weigel, Abdallah Azzeer, Christian G. Stief, Michael Chaloupka, Niels Reinmuth, Jürgen Behr, Thomas Kolben, Nadia Harbeck, Maximilian Reiser, Ferenc Krausz, Mihaela Žigman

**Affiliations:** † Ludwig-Maximilians-Universität München (LMU), Chair of Experimental Physics - Laser Physics, 85748 Garching, Germany; ‡ Max Planck Institute of Quantum Optics (MPQ), Laboratory for Attosecond Physics, 85748 Garching, Germany; § Center for Molecular Fingerprinting (CMF), 1093 Budapest, Hungary; ∥ King Saud University (KSU), Department of Physics and Astronomy, 11451 Riyadh, Saudi Arabia; ⊥ Leibniz Institute of Photonic Technology-Member of the Research Alliance “Leibniz Health Technologies”, 07745 Jena, Germany; # University Hospital of the Ludwig Maximilians University Munich (LMU), Department of Urology, LMU, 81377 Munich, Germany; 7 Asklepios, Department of Thoracic Surgery, Member of the German Center for Lung Research, DZL, Asklepios Fachkliniken München-Gauting, 82131 Gauting, Germany; 8 Department of Medicine V, LMU University Hospital, Comprehensive Pneumology Center, German Center for Lung Research, LMU, 81377 Munich, Germany; 9 University Hospital of the Ludwig Maximilians University Munich (LMU), Department of Obstetrics and Gynecology, Breast Cancer and Comprehensive Cancer Center Munich (CCLMU), LMU, 81377 Munich, Germany; 10 University Hospital of the Ludwig Maximilians University Munich (LMU), Department of Clinical Radiology, LMU, 81377 Munich, Germany

## Abstract

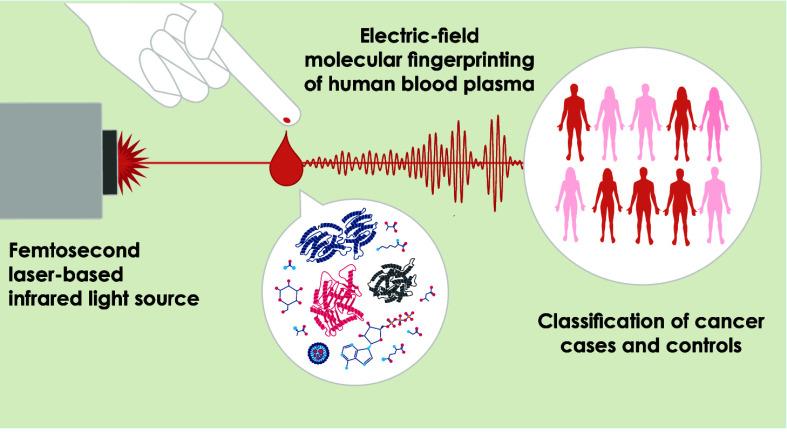

Human biofluids serve as indicators of various physiological states, and recent advances in molecular profiling technologies hold great potential for enhancing clinical diagnostics. Leveraging recent developments in laser-based electric-field molecular fingerprinting, we assess its potential for *in vitro* diagnostics. In a proof-of-concept clinical study involving 2533 participants, we conducted randomized measurement campaigns to spectroscopically profile bulk venous blood plasma across lung, prostate, breast, and bladder cancer. Employing machine learning, we detected infrared signatures specific to therapy-naïve cancer states, distinguishing them from matched control individuals with a cross-validation ROC AUC of 0.88 for lung cancer and values ranging from 0.68 to 0.69 for the other three cancer entities. In an independent held-out test data set, designed to reflect different experimental conditions from those used during model training, we achieved a lung cancer detection ROC AUC of 0.81. Our study demonstrates that electric-field molecular fingerprinting is a robust technological framework broadly applicable to disease phenotyping under real-world conditions.

## Abstract 2

Laser-based infrared molecular fingerprinting detects cancer, demonstrating its potential for clinical disease diagnostics.

## Introduction

Various human phenotypes, including diseases, are reflected in the molecular makeup of biofluids such as blood and its cell-free media like serum and plasma.^1−4^ Despite a significant medical need to complement current invasive and resource-intensive diagnostic techniques with time- and cost-effective noninvasive alternatives, a key challenge for modern omics technologies remains to achieve reproducible and robust multimolecular detection and interpretation.^4−6^ Sensitive and specific analytical methods in the fields of proteomics^6−9^ and metabolomics^10−13^ have led to the discovery of numerous molecular “biomarker candidates”. However, current omics techniques are often still limited in the range of molecular species that they can probe at once. They often require complex, target-specific preanalytical workflows for sample preparation.

There is an alternative approach known as molecular fingerprinting, where phenotype detection is based on patterns of change across the entire molecular landscape.^2,14^ If a specific pattern shows a robust correlation with a particular physiological state, it may contribute to the detection of a phenotype. Differences in the patterns of, for example, peptides and metabolites, reflected in the spectra obtained by mass spectrometry (MS)^9,15^ and nuclear magnetic resonance (NMR)^16−18^ spectroscopy, have shown potential for disease detection. Multiomics, which targets multiple molecular species,^4,19^ promises to improve diagnostics capabilities. However, such efforts also require sophisticated methods of combining different data sets.^4,5,19,20^ Broadband vibrational spectroscopy overcomes these challenges by measuring the entire molecular landscape in a single cross-molecular fingerprint as demonstrated with Fourier transform infrared (FTIR) spectroscopy.^21−25^ Numerous studies using FTIR spectroscopy have shown the potential of blood-based infrared spectroscopic molecular fingerprinting for disease detection.^22,25−32^

In conventional FTIR spectrometers driven by thermal radiation sources, minute changes in molecular absorption can be drowned out by the strong excitation background, limiting the sensitivity of the instrument.^33−35^ Laser-based spectroscopic approaches such as electric-field molecular fingerprinting (EMF) can overcome this limitation.^33,36,37^ Here, the sample is excited by an ultrashort pulse of broadband infrared light, lasting only tens of femtoseconds. After the excitation, the molecules emit their resonant vibrational response over a period extending over hundreds of femtoseconds to several picoseconds depending on dephasing times. Using nonlinear optical wave-mixing, this response can be captured in a time-resolved manner and temporally separated from the excitation, thus obtaining infrared electric-field molecular fingerprints, henceforth briefly: infrared fingerprints throughout this text. Moreover, no broadband FTIR instrument has demonstrated high-throughput capabilities so far. In contrast, the bright laser excitation in EMF lends itself to the high-throughput measurements required for screening applications.

Here, we report the first proof-of-concept biomedical application of EMF, demonstrating that the measured fingerprint patterns robustly acquire disease-specific information from liquid blood plasma. Our findings indicate that patterns in infrared fingerprints can reliably be associated with physiological states. In this initial evaluation of EMF involving sample injection automation and its first implementation in a large-scale clinical study setting, we were able to detect lung cancer in a minimally invasive manner. To evaluate the robustness of our method, we utilized an independent held-out test set designed to emulate more realistic conditions such as variations in the measurement apparatus, which can occur in real-world screening scenarios. This independent testing allows us to assess the generalizability of our technique beyond a single measurement campaign, revealing that our lung cancer detection model remains robust under realistic measurement shifts, maintaining its diagnostic performance and demonstrating its potential reliability.

## Results

### Electric-Field Fingerprinting to Profile Human Blood Plasma

Infrared vibrational spectroscopy examines the vibrational response of molecular bonds to optical excitation. It accesses the frequency, phase, and oscillator strength of the infrared-active vibrational modes specific to the molecule(s) under scrutiny, which may facilitate their identification and quantification.^38^ Unlike the continuous irradiation of the sample by an infrared source in FTIR spectroscopy, EMF employs impulsive excitation with an ultrashort laser pulse, followed by time-resolved sampling of the infrared electric field emitted by the sample.^33,36^ As a result, the coherent molecular response survives the ultrabrief excitation, and direct measurements of temporal signals free from the excitation source and its associated noise lead to improved sensitivity.^33^ For complex human biofluids, infrared electric fields emitted by different classes of molecules (e.g., proteins and carbohydrates) add up coherently to form the sample’s cross-molecular infrared fingerprint. The present study assesses the potential of EMF technology as a platform for *in vitro* blood plasma profiling in a clinical study, specifically for cancer diagnostics.

**Figure 1:**
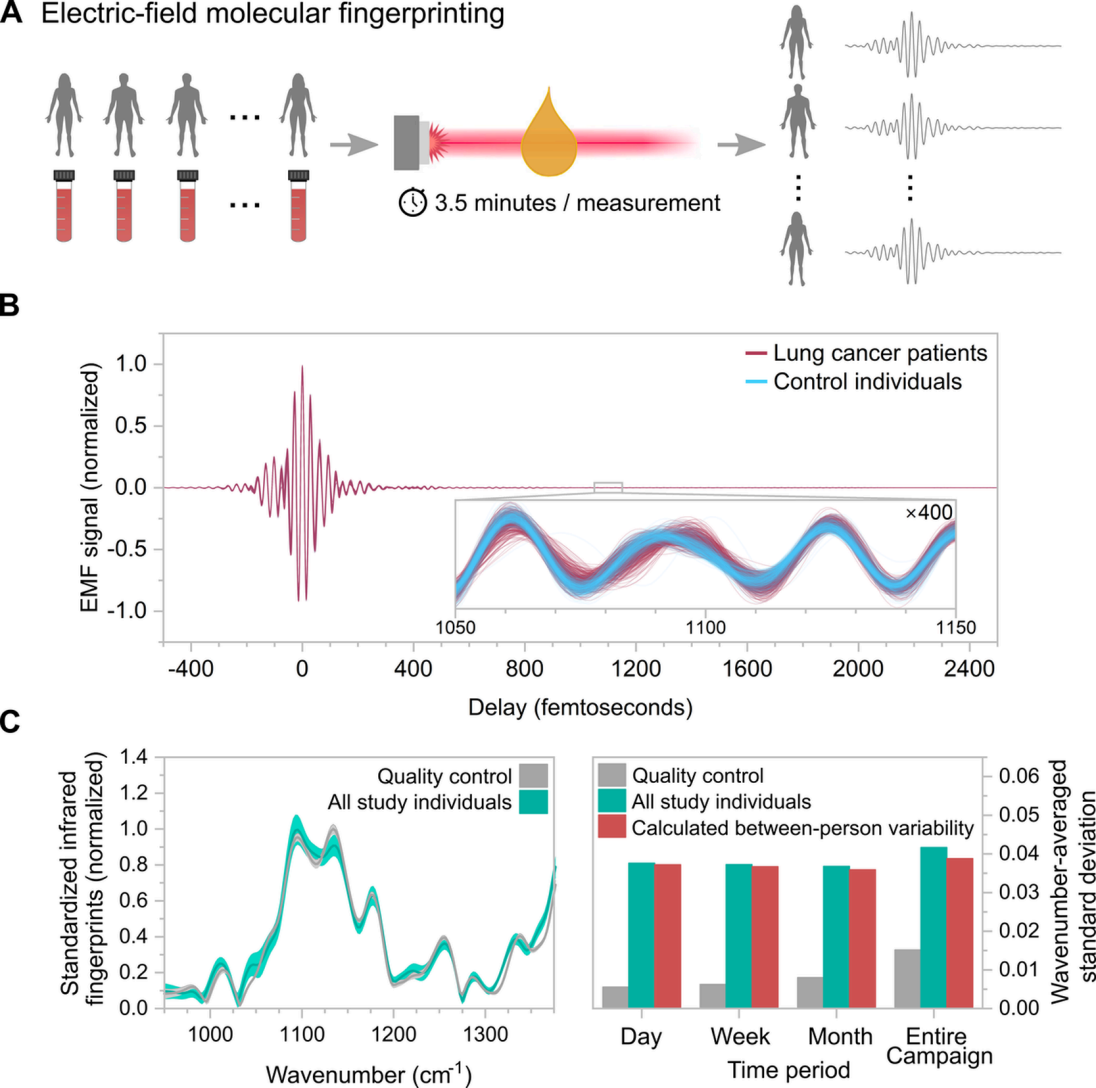
Electric-field molecular fingerprinting for *in vitro* diagnostics. (A) Simplified scheme describing the EMF process for human phenotype detection using venous blood plasma. Each individual enrolled in the clinical study was medically characterized, had venous blood sampled, and processed blood plasma sample measured using an EMF instrument resulting in an infrared electric-field molecular fingerprint. (B) Examples of infrared electric-field molecular fingerprints. The plot displays EMF signals obtained from blood plasma samples of lung cancer patients (magenta) and control individuals (light blue). The inset zooms into the EMF signals in the delay range from 1050 to 1150 fs, with the signal values amplified by a factor of 400 along the *y*-axis. (C) Reproducibility of standardized EMF measurements performed over the measurement campaign. (Left) Mean (solid line) and standard deviation (shaded region) of standardized infrared electric-field molecular fingerprints for identical quality control samples (gray) and samples from the *Lasers4Life* clinical study (cyan), acquired over the seven-month-long measurement campaign. (Right) Wavenumber-averaged standard deviation values correspond to four time scales plotted for the quality control (gray) and clinical study samples (cyan). The between-person variability (red) for each time interval was estimated as the square root of the difference between the two variances represented in cyan and gray. Details on the EMF measurement preprocessing and standardization procedure that led to the results shown here are provided in the Methods section. Figure S3 provides a comparable reproducibility analysis for spectra obtained using an FTIR spectrometer.

Figure 1(A) illustrates the experimental setup, which includes sample collection, impulsive infrared excitation, and EMF measurement of human blood plasma. Blood plasma samples from 2533 individuals were collected as part of the *Lasers4Life* clinical study and measured using the EMF instrument described in a previous publication.^37^ Each EMF measurement took 90 s and was followed by a cuvette cleaning step that lasted 2 min, adding up to a total time of 3 min and 30 s for each sample. The 90 s comprises a 40-s-long blank measurement with pure water in the cuvette, followed by sample injection and a 40-s-long measurement with the sample in the cuvette. Each 40-s-long EMF signal measurement was obtained by averaging 112,000 individual traces. The blank measurement is used to standardize our infrared fingerprints (see the Methods) by suppressing fluctuations that arise from the laser source. The measurement campaign was conducted over 73 days of operation spanning seven months. This included a 10-week time gap introduced between measurements performed on the train and test sets to simulate real-world conditions. The daily time allotted for the clinical study samples was limited due to the 2-h stabilization period required by the laser source and the time needed to measure samples not included in this study. Figure 1(B) displays infrared fingerprints normalized to their peak values plotted as a function of delay in femtoseconds, for samples of blood plasma from a representative set of lung cancer patients and control individuals, in magenta and light blue, respectively. The inset displays a magnified view of the EMF signals in the delay range from 1050 to 1150 fs.

Reproducible measurements are a prerequisite for applying the experimental approach in a medical diagnostic setting. This led us to evaluate whether the recently developed EMF technology, now integrated with semiautomated sample delivery, is sufficiently robust for cross-comparing fingerprint information over extended measurement periods as required for large clinical studies and in future health screening applications. We began by assessing the stability of the analytical approach by comparing chemically identical samples. To this end, we conducted repeated measurements on 1185 aliquots of commercially obtained pooled human blood sera, which we used as a quality control measure. To realize the background-free advantage of EMF by separating the resonant molecular signal from the impulsive excitation, we applied a time-domain filter to the measured infrared fingerprints as part of our standardization process, resembling the procedure outlined previously.^39^

The left panel in Figure 1(C) displays the mean (solid gray line) and standard deviation (gray-shaded region) of all 1185 standardized infrared fingerprints of the quality control serum samples measured throughout the campaign, normalized to the peak value within the displayed spectral range from 950 to 1375 cm^–1^, where the spectral amplitude is typically higher than 50% of the absolute maximum. Details on the EMF measurement preprocessing and standardization procedure that led to the results shown in this panel are provided in the Methods section. The spread in repeated measurements of these identical samples represents the experimental uncertainty in our EMF technique due to variations in the automated sampling procedure, fluctuations in the laser source, and the EMF detection. To compare this with the biological variability in blood plasma derived from different individuals of the study, we display the same measures in cyan for the 2533 plasma samples collected from participants of the *Lasers4Life* clinical study (see the Methods for details on sample selection and cohort design). The right panel shows the average value of the standard deviation, calculated over all wavenumbers from 950 to 1375 cm^–1^. The wavenumber-averaged standard deviation was calculated for both the quality control (gray) as well as the *Lasers4Life* study samples (cyan) for measurements acquired over time scales of a day, a week, a month, and the entire measurement campaign, which lasted seven months.

Although the experimental variability in the quality control measurements increased over the extended period of measurement comparison, the value remained considerably lower than the variability between different individuals within the study, underscoring the potential of employing field-resolved spectroscopy for large-scale analyses spanning several months. We estimated the standard deviation corresponding to the between-person variability in the standardized EMF signals (red bars) as the square root of the difference between the variances corresponding to the study individuals and the quality control samples. This corresponds to the square root of the difference between the squares of the gray and cyan plots. Although reproducibility in this initial EMF implementation is not yet at the level of advanced FTIR spectrometers (see Figure S3), ongoing developments in EMF instrumentation are expected to significantly reduce instrument noise (gray bars), thereby enhancing EMF sensitivity.^40^

### Clinical Study Setting

**Figure 2:**
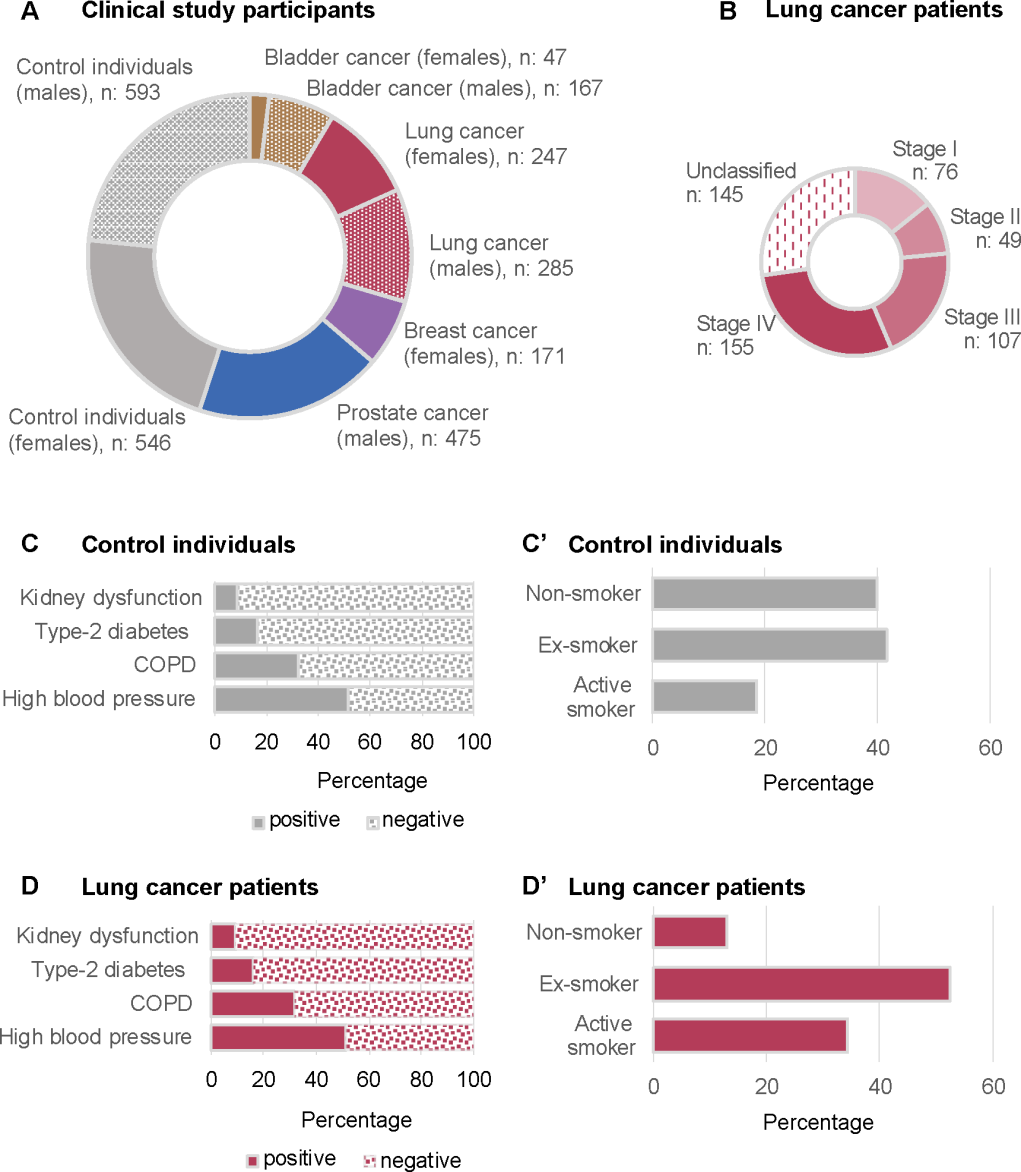
Detailed breakdown of the *Lasers4Life* clinical study cohort. This figure presents the distribution of 2533 study participants. (A) Pie chart categorizes participants by cancer status, specific cancer types, and sex. (B) Breakdown of lung cancer patients by stage. (C and C′) Distribution of key characteristics, including comorbidities and smoking status, within the control group. (D and D′) Distribution of key characteristics, including comorbidities and smoking status, within the lung cancer group. Additional information on age and BMI distributions by cancer group is provided in Figure S1.

The capacity of EMF to aid cancer diagnostics was tested in the multicentric *Lasers4Life* clinical study conducted in the Munich area, where the study participants were divided into case-control group pairs of therapy-naïve cancer patients (with cancer of either the lung, prostate, breast, or bladder) and asymptomatic control individuals. Figure 2 visually represents the cohort statistics. Panel (A) shows a breakdown into the four different cancer groups and the group of noncancer control individuals, as well as based on sex. The set containing all study participants (patients and reference individuals) was randomly split into training and test sets. The training set altogether consisted of 2104 individuals, corresponding to approximately 80% of the total number of participants. The EMF measurements of these individuals were conducted in a fully randomized manner over 19 weeks. The remaining 20% (429 individuals) constituted the test set, which was measured in randomized order over 2 weeks, following a 10-week gap that was introduced to ensure robust testing considering drifts in spectrometer performance. During this gap period, the EMF measurements of samples that are not part of the *Lasers4Life* clinical study cohort were performed using the instrument. Case-control group pairs were created and utilized within the training data set to train binary classification models tailored to target medical questions. To account for potential confounding factors, statistical matching based on age and sex was employed.

Throughout the clinical study, venous blood was processed to plasma according to previously defined standard operating procedures to minimize preanalytical errors.^27^ An automated sample delivery system was applied for transmission mode spectroscopic measurement. The samples were excited by broadband (910–1530 cm^–1^ at −20 dB intensity) mid-infrared laser pulses with a duration of 60 fs (full width at intensity half-maximum), and the molecular response was recorded over 40 s with dual-oscillator electro-optic sampling.^37^

In the first step, using the training set, we assessed the feasibility of EMF to distinguish therapy-naïve lung, prostate, breast, and bladder cancer patients (cases) from age- and sex-matched asymptomatic control individuals (controls). The acquired infrared fingerprints were used to train machine learning models to perform binary classification of the samples into cancer and non-cancer reference groups. Model training was performed by applying a logistic regression algorithm to standardized infrared fingerprints^37,39^ of the training set. For initial performance evaluation, 10-fold cross-validation was used, repeated 5 times with randomization. The classification performance was assessed by evaluating the area under the receiver operating characteristic (ROC) curve (AUC). The optimized classifiers were then tested on the independent held-out test data sets (Section 2.4). The test sets were not statistically matched. Matching in terms of age and sex was performed only on the disease-specific training data sets to avoid introducing bias into the classification models.

Due to the high efficiency of lung cancer classification compared to asymptomatic control individuals (shown in the following subsections), we extended our analysis to examine comorbidities and disease progression within the training set. Figure 2(B) illustrates a distribution of lung cancer stages in the study. Figures 2(C) and 2(D) show the prevalence of four chosen conditions, namely kidney disease, type-2 diabetes, chronic obstructive pulmonary disease (COPD), and high blood pressure among the group of control individuals and that of lung cancer patients, respectively, while Figures 2(C′) and 2(D′) display the smoking status of participants in the two groups.

### Electric-Field Fingerprinting Platform for Analyzing Molecular Signatures of Four Common Cancers

**Figure 3:**
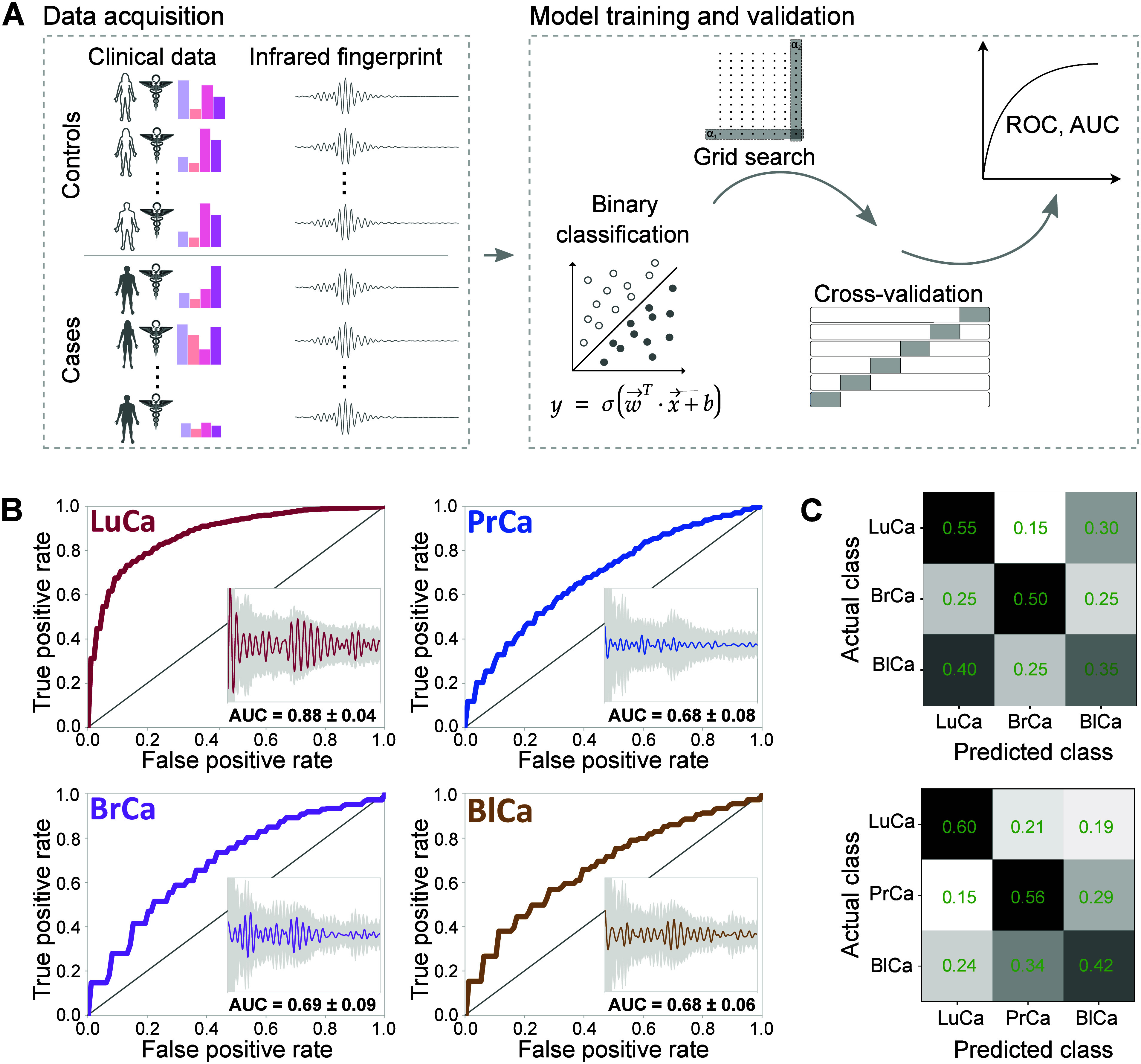
Electric-field resolved fingerprinting for *in vitro* detection of four common cancers. (A) Schematic of the machine learning pipeline used to generate mean ROC curves, involving binary classification models trained to distinguish cancer cases from nonsymptomatic controls. Model training was performed using logistic regression within a nested cross-validation framework (see the Methods for details). (B) Mean ROC curves illustrating the detection performance for each cancer type. Insets display the mean difference in EMF signals between cancer patients and control individuals (solid line), along with the standard deviation in the EMF signal of the corresponding controls (gray-shaded region). The *x*-axis represents the delay, ranging from 500 to 1200 fs. The *y*-axis scale is identical across all four insets, ensuring direct comparability. Mean test AUC values from the cross-validation are 0.88 ± 0.04 for lung cancer, 0.68 ± 0.08 for prostate cancer, 0.69 ± 0.09 for breast cancer, and 0.68 ± 0.06 for bladder cancer. (C) Multiclass classification of different cancer types. Confusion matrices show classification results for lung, breast, and bladder cancers in a matched female cohort (upper plot) with an overall model accuracy of 0.48 ± 0.11, and for lung, prostate, and bladder cancers in a matched male cohort (lower plot) with an overall model accuracy of 0.53 ± 0.03. Further details on the demographic characteristics of the matched case-control designs used in this analysis can be found in Tables S1 and S2.

We evaluated the ability of EMF to detect four cancer entities, lung, prostate, breast, and bladder cancer, when compared to age and sex-matched nonsymptomatic control individuals each. Figure 3(A) outlines the workflow where patient medical information and fingerprint measurements from the training data set were applied to train and evaluate logistic regression models within a nested cross-validation scheme, as detailed in the Methods section. The corresponding ROC curves for each cancer type, representing the average ROC calculated through cross-validation within the training data, are shown (Figure 3(B)). Additionally, the four insets show the mean difference in the EMF signals obtained from blood plasma samples between cancer and control groups of individuals. The analysis indicates a test AUC upon cross-validation of 0.88 ± 0.04 for lung cancer detection, while AUC values for the other cancer types are lower and range between 0.68 and 0.69. These AUCs are closely tied to the effect size, which is the ratio of the differential signal magnitude caused by the condition to the spread of the control measurements (as shown in the insets). A future reduction in instrument noise is expected to increase the effect size and thus improve classification performance. This stronger result for lung cancer detection is consistent with the fact that lung tumors generally grow more rapidly than many other types of cancer, though growth rates vary widely across cancer subtypes and organs. Another possible explanation is that lung tumors may release more metabolic and cellular products into the bloodstream, given the closer proximity and exchange with the circulatory system.

To better detect spectroscopic aberrations of lung cancer, whose aggressive nature underscores the importance of early detection and timely treatment, we further investigated the influence of demographic parameters, such as sex, age, and BMI, on the trained classification models. Our results indicate that these demographic parameters do not significantly affect model performance (Figure S2), supporting the robustness of the approach across different populational substrata. Observed trends, however, suggest a slightly more efficient detection of lung cancer in individuals with lower BMI.

To test whether EMF signals are specific to different cancer entities, we explored the classification of cancer types within a balanced cohort of cancer patients, excluding control individuals. We created sex-stratified cohorts, each comprising three cancer types, with subgroups statistically matched based on age. Multiclass classification algorithms were then trained to predict the cancer type within cross-validation. Figure 3(C) presents the resulting confusion matrices. For the female cohort, the model achieved an overall accuracy of 0.48 ± 0.11, while the male cohort achieved an accuracy of 0.53 ± 0.03. These results are significant, as random chance prediction would yield an accuracy of only 0.33, underscoring the capacity of electric-field fingerprints to capture cancer-specific signals.

### Testing EMF Performance under Nonidentical Conditions

To ensure the robustness and generalizability of our machine learning models, it is crucial to perform independent testing using a held-out test set, validating the performance and reliability of our models in realistic scenarios. To address this, we conducted an independent measurement campaign 10 weeks after the initial experimental measurements were used for model training. This method surpasses the traditional approach of reserving a subset of data for testing by providing a statistically independent test set with data that fall outside the training distribution, thus offering a more realistic evaluation.

**Figure 4:**
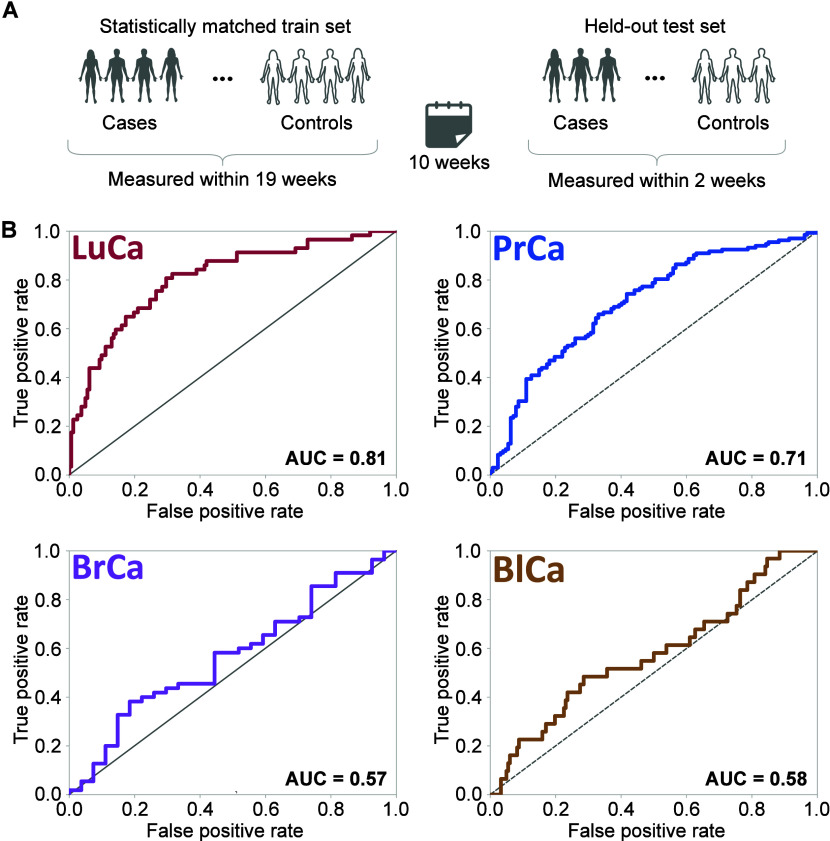
Performance of EMF-based models in predicting four common cancers on an independent test set, obtained from a separate measurement campaign conducted 10 weeks after the original campaign used for model training. (A) Overview of the measurement campaign: The total population was randomly split into a training set (80%) and an independent held-out test set (20%), with measurements for each set conducted in two separate campaigns, spaced 10 weeks apart. (B) Receiver operating characteristic (ROC) curves demonstrate the performance of cancer-specific binary classification models on the independent held-out test sets. Detailed demographic characteristics of these test sets are provided in Table S1.

The model’s performance slightly decreased when applied to the independent test set. For lung cancer, the AUC dropped from 0.88 to 0.81. Similar declines were observed for the other three cancer entities, with the most significant drop seen in breast cancer, where the AUC fell to around 0.5, rendering the current EMF instrument incapable of detecting breast-cancer-specific signals in the independent cohort (Figure 4). These discrepancies are expected due to measurement device drifts and differences in cohort characteristics. Such variations are common in real-world scenarios and provide more realistic performance estimates.

The present work marks the first evidence that EMF signals of blood plasma can reliably capture signals linked to at least three types of cancer (lung, prostate, and bladder). Already in its first implementation, the cancer diagnostic performance with EMF is comparable to that of FTIR fingerprinting (Table S9). These promising findings suggest that further technological improvements, such as expanded spectral coverage and enhanced stability, could significantly boost EMF’s diagnostic potential.

Given EMF’s strong performance in lung cancer detection, the remaining analyses focus on its utility in improving lung cancer diagnostics, including aspects of cancer progression and related comorbidities. For comprehensive evaluation, all further analyses are conducted using the full training data set with cross-validation, rather than the held-out test set.

### Performance of EMF Correlates with Lung Cancer Staging

**Figure 5:**
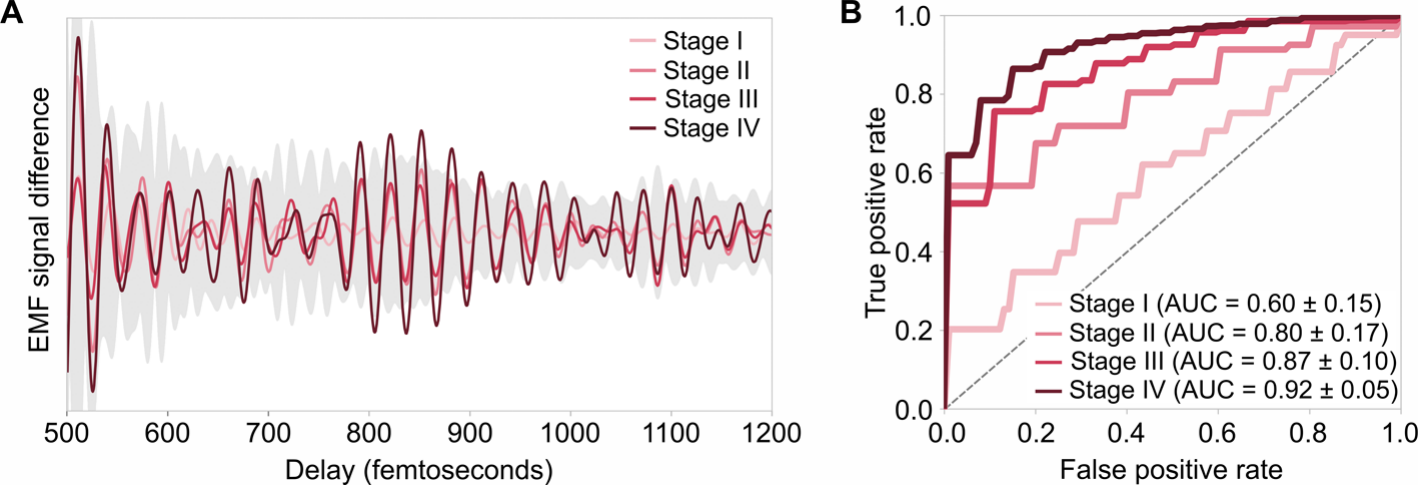
Lung cancer progression (in terms of TNM staging), as reflected by EMF. (A) The mean difference in measured plasma EMF signals between cancer patients and control individuals (solid line) and the standard deviation in the EMF signal for the control individuals (gray-shaded region), plotted against the time delay ranging from 500 to 1200 fs for better visibility, stratified by lung cancer stage. (B) Average ROC curves (from nested cross-validation) for classification models applied to different case-control groups, stratified by the TNM staging of lung cancer cases. Demographic characteristics of the case-control designs used in this analysis are detailed in Table S3.

To explore the potential of minimally invasive infrared diagnostics for early stage lung cancer detection, which could enhance treatment options, we evaluated classification models across different lung cancer case-control groups stratified by tumor, node, metastasis (TNM) staging, following the TNM Classification of Malignant Tumors (Union for International Cancer Control (UICC)).^41^Figure 5(A) illustrates the difference between EMF signals from lung cancer patients and control individuals for four case-control designs, stratified by lung cancer stage. We observe that signals monotonically increase with disease progression, indicating a “dose–response” effect, where higher tumor progression corresponds to stronger signals. This finding provides strong evidence that the differential EMF signals are indeed tumor-specific and in agreement with our previous FTIR spectroscopic examinations.^27^ The corresponding classification performance is depicted in Figure 5(B), assessed through cross-validation within the training set. This analysis reveals a significant influence of the disease stage on classification accuracy and the capacity of EMF to detect the disease. The observed dose–response relationship here further underscores the tumor-specific nature of the captured EMF signals.

A stage-wise comparison of EMF- and FTIR-based model performance is detailed in Table S10. Notably, both methods yield near-identical results regarding stage-wise ROC AUC values and average spectrally resolved effect sizes across wavenumbers that significantly contribute to class separation.

### Impact of Physiological Comorbidities on Lung Cancer Detection Efficiency

**Figure 6:**
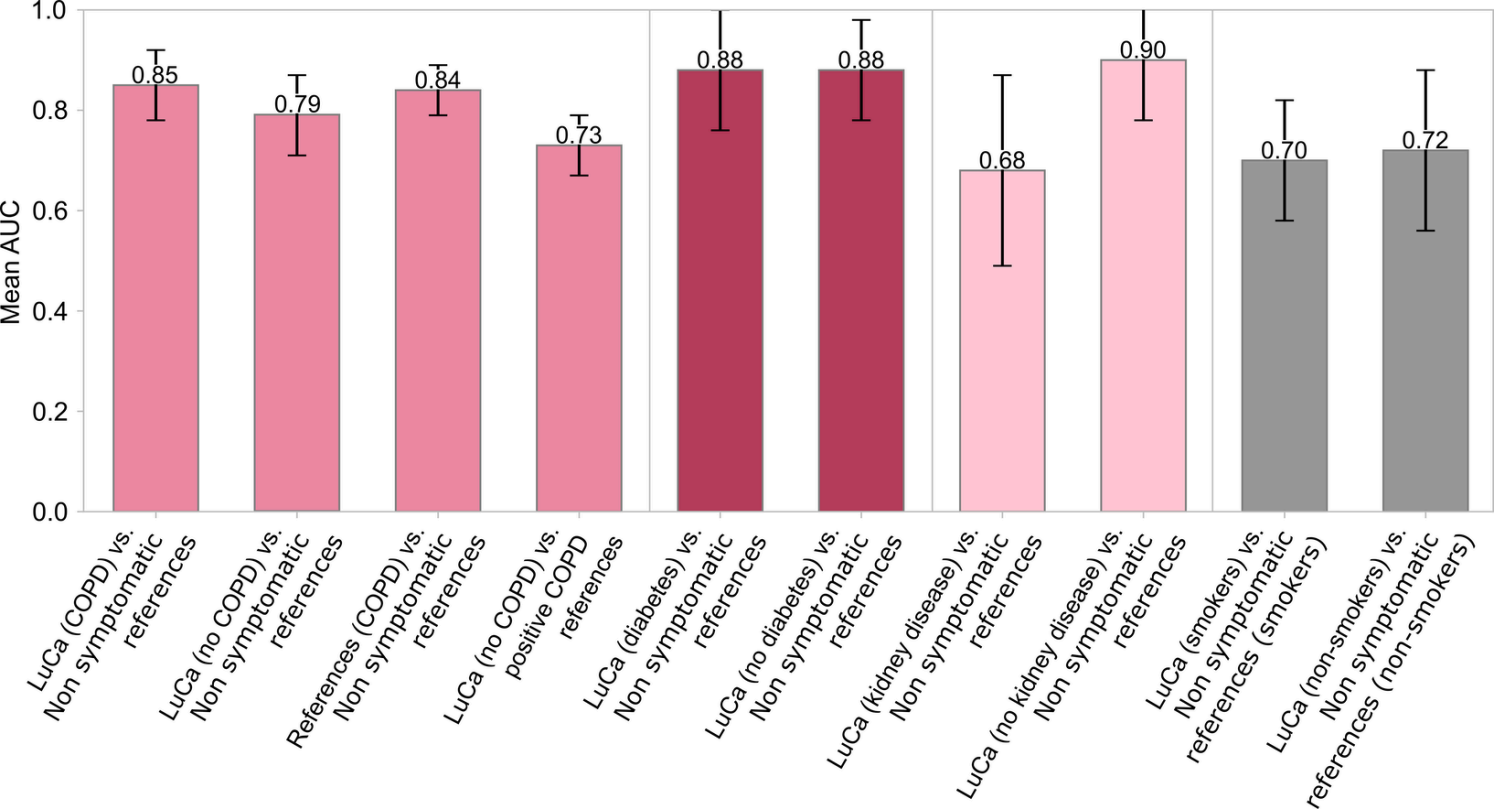
Effect of physiological conditions and smoking status on lung cancer detection accuracy using electric-field fingerprinting. Bars represent cross-validated ROC AUC values for EMF-based binary classification models trained on case-control designs with lung cancer cases stratified by the presence or absence of relevant comorbidities. Detailed demographic characteristics of the matched cohorts used in this analysis are provided in Tables S4 and S5.

Cancers are commonly accompanied by one or more chronic conditions (comorbidity/multimorbidity) at the time of diagnosis, which can affect its detection and prognosis.^42^ To evaluate the capacity of infrared fingerprinting under physiologically realistic conditions, we directly tested whether pre-existing chronic conditions limit its functionality. Chronic obstructive pulmonary disease (COPD), a chronic lung condition marked by obstructed airflow and breathing difficulties, often coexists with nonsmall cell lung cancer (NSCLC), particularly in smokers.^43^ Since molecular changes in blood plasma due to COPD could impair lung cancer detection, we systematically examined its impact on EMF-based detection models. Figure 6 presents the influence of COPD and other comorbidities on these models. The first 2 bar show ROC AUC values for matched case-control data sets for cases stratified by COPD status. Cases included in the two bars were matched by cancer stage to avoid confounding factors related to disease progression. We observed a difference in AUC values, indicating a more efficient detection performance in populations without COPD as a comorbidity. The third bar displays the ROC AUC value for detecting COPD among cancer-free individuals, showing a high mean AUC of 0.84, confirming COPD’s detectability of COPD with our fingerprinting approach. The fourth bar shows that EMF-based models can effectively differentiate between lung cancer and COPD patients, achieving a mean AUC of 0.73. Beyond COPD, we further assessed whether type-2 diabetes or kidney disease could impact the detection of lung cancer. The fifth and sixth bars of this plot show the corresponding ROC AUC values for cases that are positive and negative in type-2 diabetes mellitus, respectively, matched by lung cancer stage. We found that type-2 diabetes did not impact lung cancer detection, an important finding for application as type-2 diabetes mellitus is a very common condition. Kidney dysfunction commonly co-occurs with lung cancer, so it is also important to evaluate its potential effect on the capacity of EMF-based models to detect lung cancer. Conversely to COPD, chronic kidney disease significantly affected lung cancer detection models, potentially hindering accurate lung cancer diagnosis. In addition to comorbidities, we also tested the influence of smoking status on the lung cancer detection efficiency. The last two bars of Figure 6 compare the resulting ROC AUCs when stratifying individuals in terms of smoking status and show no significant difference. While detecting, managing, and taking into account possible comorbidities are crucial in medical test development, plasma-based EMF grossly shows robust capabilities at the heterogeneous group level, warranting consideration for *in vitro* diagnostics, pending further clinical validation in future independent populations and clinical studies.

## Discussion

EMF enables the comprehensive profiling of molecular mixtures in human blood plasma. This study establishes the technique as a promising candidate for an *in vitro* diagnostic application through a large-scale case-control clinical study. Focusing on cancer detection, we demonstrate how field-resolved spectroscopy allows for phenotype inspection independently of the nature of a phenotype or molecular composition. Previously reported experiments on aqueous solutions of organic molecules^33^ show an enhanced sensitivity for EMF compared to conventional FTIR spectroscopy. The demonstrated higher inherent sensitivity of EMF as compared to conventional time-integrated spectroscopies, along with a future extension of spectral coverage, holds promise for significant improvement of the classification efficiencies demonstrated here with first-generation EMF instrumentation. At its current stage of development, EMF stands at par with FTIR spectroscopy (see comparisons in Tables S9 and S10).

To robustly evaluate the reliability of the experimental technique, we ensured that the human blood sample collection, plasma processing, and preanalytical workflows adhered to previously established standards,^23^ and processed the measured EMF signals as per a carefully designed standardization procedure.^39^ We report the stability and reproducibility of our approach over extended periods and larger-scale measurement paradigms. The analysis of 1185 quality control samples and blood plasma samples from 2533 different individuals confirmed the robustness of the setup over seven months of operation. Despite slight increases in measurement variability over time, the overall fingerprinting variation consistently remained lower than the biological variability between different individuals. With this as a promising starting point, we expect future versions of EMF instruments with improved stability and noise characteristics^44^ to surpass the diagnostic performance of conventional fingerprinting.

We recorded infrared electric-field molecular fingerprints of blood plasma samples from individuals with lung, prostate, breast, or bladder cancers and trained a multiclass classifier using the data. Our results reveal the ability of the approach to distinguish patients with different cancer types from each other, supporting the specificity of infrared fingerprints, distinct for each of the studied cancers. We then analyzed the fingerprints corresponding to each cancer type separately and compared them to those from a matched control group to train a binary classification model. We evaluated our model on a held-out data set of blood samples from different individuals, measured in a separate measurement campaign which was started several weeks after the completion of the first campaign. This approach allowed us to assess how the model performs on data obtained under experimental conditions different from those used during model training. Although a decrease in performance was observed in comparison to cross-validation, the AUC for lung cancer detection remained robust at 0.81. The observed discrepancies, particularly in the capacity to detect the other three cancer entities, highlight the need for improving the reproducibility of EMF measurements and for further validation of this approach in additional patient populations. Encouragingly, the detection of lung cancer across different stages revealed a dose–response relationship. In particular, we observed that stronger EMF signals are associated with more advanced disease stages, consistent with our previous FTIR findings.^27^

Given that cross-molecular fingerprint information remains stable over several years^23^ and that proper longer-term sample storage preserves infrared signals of blood plasma,^25^ infrared fingerprinting carries the capacity to contribute to medical diagnostics. In the context of lung cancer detection, while abnormal kidney function impacted model accuracy, the models effectively distinguished lung cancer patients from matched individuals, despite common comorbidities such as COPD and type-2 diabetes mellitus. This robustness further highlights the clinical utility of the approach, either complementary to golden-standard medical diagnostics or as a novel tool for disease risk stratification.

Newly exploring the phenotype diagnostic capacities of EMF, it is encouraging to observe the stability of the new technology across extended experimental periods combined with reproducibility in held-out test sets for three out of four tested cancers, even at this early stage of technological development. This is particularly significant given that the current EMF instrument covers only a small fraction of the molecular fingerprinting region of the entire electromagnetic spectrum. Further technological advancements leading to EMF instruments with a broader spectral coverage^40,45,46^ hold promise for capturing even more molecular information. Interferometric subtraction of EMF signals could enhance detection sensitivity by suppressing the technical noise arising from the impulsive excitation pulse.^47^ The rapid acquisition capability of the current EMF instrument,^37^ which captures thousands of EMF traces per second, also suggests potential for applications beyond plasma fingerprinting, such as the real-time tracking of reaction dynamics,^48−50^ in-line infrared spectroscopic monitoring of chromatographic processes,^51^ and label-free flow cytometry.^52^

Another significant area of technological advancement is the development of new laser sources. The advent of powerful and widely tunable quantum cascade lasers (QCLs), which emit radiation directly in the mid-infrared spectral region, has profoundly impacted research in biomedical spectroscopy.^53^ With output power in the milliwatt range—orders of magnitude higher than conventional thermal sources of infrared radiation—QCLs as well as the ultrafast-laser-based technique described in this work enable the probing of liquid biological samples over larger sample thicknesses.^54,55^ The application of new spectroscopic methods in combination with machine learning to effectively analyze spatially resolved infrared spectral images in histopathology has gained significant attention^56^ due to their potential to aid the medical diagnostic process. Recent studies have shown increasing medical explainability by correlating infrared molecular fingerprints with conventional clinical chemistry measurements.^25^ Spectral changes in infrared fingerprints are being understood better with the help of other omics approaches^24^ and additional preanalytical techniques that decompose the molecular complexity of biological matrices.^57^ Other developments have focused on computationally modeling the infrared absorption spectra of proteins^58^ as well as the energy transfer mechanism behind electric-field molecular fingerprints.^59^ Together, these developments could push the boundaries of infrared spectroscopy for biomedical applications.

In conclusion, the current findings provide compelling evidence underscoring the potential of electric-field molecular fingerprinting for minimally invasive disease detection. This new technology, already performing on par with conventional FTIR spectroscopy, achieves this through our technological improvements like standardized sample handling and improved instrument stability along with a new rapid-scanning technique and effective data processing. Future enhancements, such as broader spectral coverage,^40,46^ increased detection sensitivity and specificity,^44^ multidimensional measurements,^60^ and interferometric subtraction,^47^ could further boost biomedical potential. Expanding clinical studies to larger cohorts, focusing on early disease states and independent clinical testing, and exploring various disease phenotypes and their combinations will be crucial for developing a reliable diagnostic platform to improve cancer outcomes.

## Methods

### Clinical Study Participants

We conducted a multicentric, observational study involving participants with four types of cancer (lung, bladder, breast, and prostate), as well as asymptomatic volunteers serving as control subjects. Informed written consent was obtained from all participants under research study protocol 17-182. The blood samples of lung cancer patients were derived from the Asklepios biobank of lung diseases under project 333-10 and study protocol 17-141. Both research protocols were approved by the Ethics Committee of the Ludwig-Maximilians-Universität (LMU) of Munich. Our studies comply with all relevant ethical regulations and were conducted according to Good Clinical Practice (ICH-GCP) and the principles of the Declaration of Helsinki. The clinical trial is registered (ID DRKS00013217) with the German Clinical Trials Register (DRKS). Subject recruitment and sample collection were conducted at the following clinical centers of LMU University Hospital, Munich: the Department of Medicine V, the Department of Urology, and the Department of Obstetrics and Gynecology. Additional study sites included the Asklepios Clinic in Gauting and the Comprehensive Pneumology Centre (CPC) in Munich, both in Germany. Analyses focused on case subjects with clinically confirmed carcinoma of the lung, bladder, breast, or prostate who had not yet received any cancer-related therapy and had no history of other cancer occurrences. Healthy controls were asymptomatic individuals with no history of cancer and no cancer-related diseases and were not under any medical treatment. Figure 2 shows a detailed breakdown of the study participants. Cancer cases were compared to healthy individuals matched for sex and age (Supplementary Tables). In total, 4016 therapy-naive individuals, either cancer-free or diagnosed with one of the four studied cancer types, were recruited under the *Lasers4Life* study framework. After statistical matching and removal of outliers, the final cohort analyzed in this study consisted of 2533 participants.

### Blood Sample Collection and Preparation

Blood plasma samples were collected, processed, and stored following established standard operating procedures.^23,27^ Blood draws were performed using 21G Safety-Multifly needles (Sarstedt) into 4.9 mL plasma tubes, centrifuged at 2000 × *g* for 10 min at 20 °C, aliquoted, and frozen at −80 °C within 3 h of collection. Before analysis, all samples were thawed, further aliquoted, and refrozen at −80 °C to maintain a consistent number of freeze–thaw cycles. Before measurement, plasma aliquots were thawed at room temperature, shaken for 30 s, and centrifuged again at 2000 × *g* for 10 min. To avoid systematic bias, samples were measured in a random order. Quality control (QC) samples from pooled human plasma (BioWest, Nuaillé, France) were measured after every five samples to monitor and track experimental errors (Supporting Information Section 7). Additionally, dimethyl sulfone (DMSO_2_, 10 mg mL^–1^) was used as a second QC sample. Each measurement sequence comprised 25 samples, 6 QC sera, and 1 DMSO_2_ sample, resulting in a total measurement time of approximately 2 h.

### EMF Measurements

Electric-field molecular fingerprinting measurements were conducted using a field-sensitive spectrometer described in a previous work.^37^ An automated liquid sample handler and a commercial autosampler (Clade GmbH, Germany) were employed for efficient sample delivery into a flow-through cuvette and cuvette cleaning. Each plasma sample measurement was preceded by a reference measurement on pure water, followed by automatic cuvette cleaning to prevent residue carryover. EMF traces from both reference and sample measurements spanned an optical delay range of 6 ps, corresponding to a spectral resolution of 2.8 cm^–1^, with a measurement time of 40 s each. Including the time needed for sample exchange and cuvette cleaning, the total time required to measure a single plasma sample was approximately 3.5 min.

### Preprocessing and Standardization of Electric-Field Molecular Fingerprinting Measurements

The EMF signals, acquired at a rate of 2800 traces per second, were calibrated, interpolated to a common delay axis, and averaged to obtain a single trace with the EMF signal as a function of delay in femtoseconds for each 40-s-long measurement, similar to the traces shown in Figure 1(B). The preprocessing steps are described in detail in ref^37^. Each sample EMF signal is accompanied by an EMF measurement of pure water, which is used to standardize the measurements and cancel out fluctuations in the intensity and phase of the laser pulses from measurement to measurement. When carrying out EMF measurements with a femtosecond excitation pulse, the intensity and phase distribution of the excitation pulse affect the waveform describing the coherent response of the sample. We use a time-domain filter at 600 fs after the peak of the excitation pulse, making the resulting signal nominally excitation-independent and comparable to fingerprints measured with other devices, including widely prevalent FTIR spectrometers. The standardization procedure has been described in a previous work.^39^ The standardized fingerprints constitute the input data sets for subsequent machine-learning-based classification analyses.

### Statistical Methods

#### Outlier Detection

After collecting the entire data set, outliers were identified and removed using the Local Outlier Factor (LOF) method, as implemented in Scikit-Learn (v.1.1.3).^61^ LOF, which is based on k-nearest neighbors, is well-suited for moderately high-dimensional data and effectively eliminates samples exhibiting spectral anomalies. This procedure led to the removal of 46 spectra, which were excluded before the matched cohorts used in the study.

#### Statistical Matching

To achieve a covariate balance between the case and control groups in the study design, we employed optimal pair matching using the Mahalanobis distance within propensity score calipers.^62^ This implementation was carried out in R (v. 3.5.1).

#### Machine Learning and ROC Curves

Classification models were developed using Scikit-Learn (v.1.1.3),^61^ an open-source machine learning framework in Python (v.3.9.13). Binary classification models were trained using logistic regression. Performance evaluation on the training data set was conducted by using a nested cross-validation approach. Hyperparameter optimization was performed through a 5-fold grid search cross-validation nested within a repeated stratified 10-fold cross-validation with five repetitions. The results are visualized through ROC curves. The cross-validation outcomes are reported as descriptive statistics, specifically the mean and standard deviation of the resulting distribution of AUC values along with mean ROC curves. Classification models were trained and applied to the corresponding test sets based on the four main training sets (one per cancer type). The performance was evaluated by using ROC curves.

## Supporting Information Available

Detailed tables and additional figures describing the clinical study cohort used in the analysis; ROC curves summarizing the performance of EMF-based lung cancer detection across various demographic groups; and a comparative analysis between EMF and conventional FTIR fingerprinting (PDF)
Transparent Peer Review report available (PDF)
