# Supplementary material for: Electric-Field Molecular Fingerprinting to Probe Cancer
Source: ACS Cent Sci. 2025 Apr 9;11(4):560–73. doi: 10.1021/acscentsci.4c02164 (PMC12022918; doi:10.1021/acscentsci.4c02164)
Supplement: Supplementary file 1 — oc4c02164_si_001.pdf [file oc4c02164_si_001.pdf]

Supporting information for "Electric-field molecular fingerprinting to probe cancer"

Kosmas V. Kepesidis, Philip Jacob, Wolfgang Schweinberger, Marinus Huber, Nico Feiler, Frank Fleischmann, Michael Trubetskov, Liudmila Voronina, Jacqueline Aschauer, Tarek Eissa, Lea Gigou, Patrik Karandušovsky, Ioachim Pupeza, Alexander Weigel, Abdallah Azzeer, Christian G. Stief, Michael Chaloupka, Niels Reinmuth, Jürgen Behr, Thomas Kolben, Nadia Harbeck, Maximilian Reiser, Ferenc Krausz and Mihaela Žigman

This supplementary file provides detailed tables and additional figures describing the clinical study cohort used in the analysis. It also includes ROC curves summarizing the performance of EMF-based lung cancer detection across various demographic groups and a comparative analysis between EMF and conventional FTIR fingerprinting.

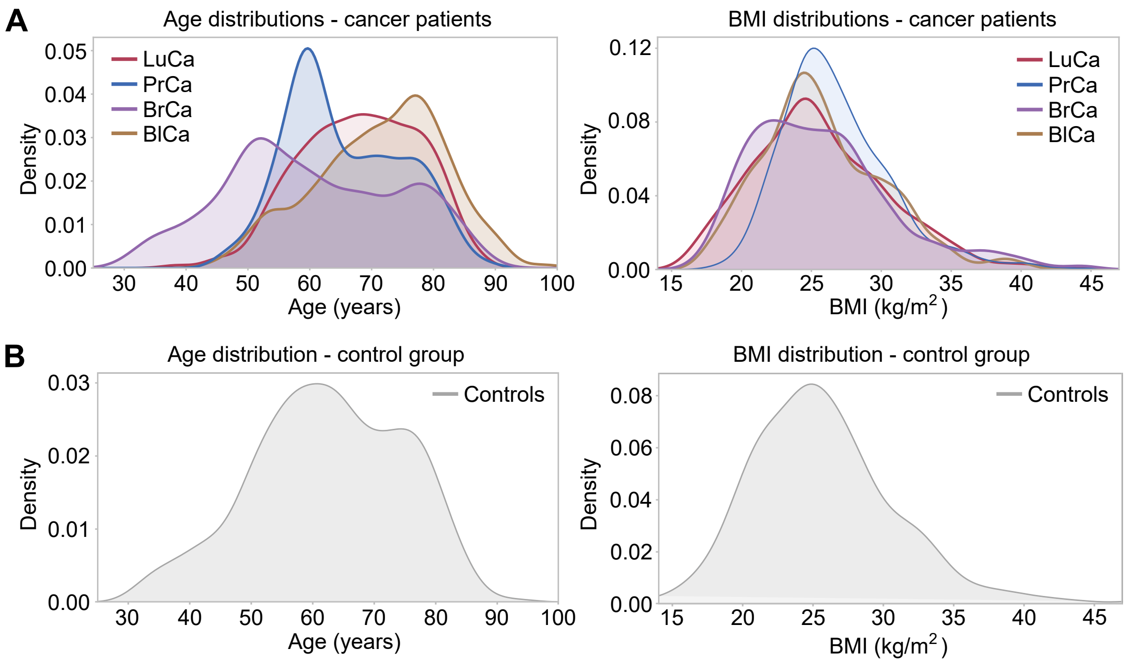

Supplementary Figure 1: Expanded cohort details, providing supplementary information to Figure 2 in the main text. **(A)** Density plots showing the age and BMI distributions within the four cohorts of cancer patients. **(B)** Density plots depicting the age and BMI distributions for the control group cohorts.

Group	# Individuals	Age (years)	% Female	BMI (kg/m^2)
Lung Cancer Training Cohort				
Cases	471	68 ± 9	46	26 ± 5
Controls	471	62 ± 10	59	26 ± 5
Prostate Cancer Training Cohort				
Cases	296	64 ± 10	0	27 ± 4
Controls	296	59 ± 15	0	27 ± 5
Breast Cancer Training Cohort				
Cases	144	60 ± 14	100	26 ± 5
Controls	144	60 ± 13	100	26 ± 6
Bladder Cancer Training Cohort				
Cases	183	72 ± 10	22	26 ± 4
Controls	183	71 ± 9	20	27 ± 5

Group	# Individuals	Age (years)	% Female	BMI (kg/m^2)
Lung Cancer Test Cohort				
Cases	57	68 ± 9	42	26 ± 6
Controls	162	66 ± 10	28	27 ± 5
Prostate Cancer Test Cohort				
Cases	132	67 ± 9	0	27 ± 4
Controls	127	67 ± 9	0	27 ± 4
Breast Cancer Test Cohort				
Cases	27	60 ± 14	100	25 ± 5
Controls	55	63 ± 13	100	26 ± 5
Bladder Cancer Test Cohort				
Cases	31	69 ± 10	26	25 ± 5
Controls	182	66 ± 11	30	27 ± 5

Supplementary Table 1: Upper sub-table: Characteristics of the matched case-control design used for training tests in model development and the primary classification analysis presented in Figure 3. Lower sub-table: Characteristics of the held-out test sets used for validating model performance shown in Figure 4.

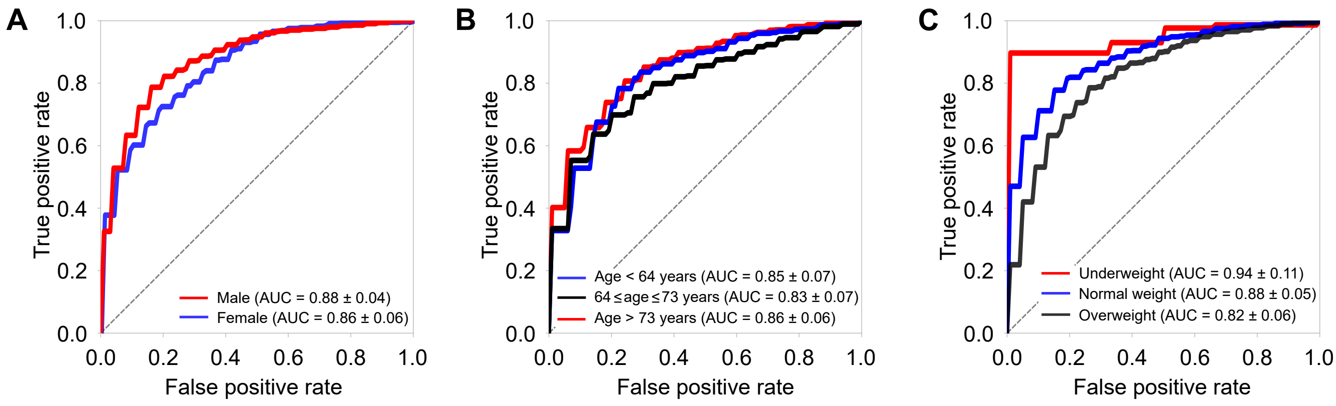

Supplementary Figure 2: ROC curves for distinguishing lung cancer from healthy control individuals across different demographic groups based on sex, age, and BMI. Details on the characteristics of the held-out test sets used in this analysis are provided in Supplementary Tables 6, 7, and 8.

Group	# Individuals	Age (years)	BMI (kg/m^2)
Male Cancer Cohort			
Lung cancer	144	71 ± 10	26 ± 4
Prostate cancer	144	71 ± 10	26 ± 4
Bladder cancer	144	72 ± 10	26 ± 4
Female Cancer Cohort			
Lung cancer	40	72 ± 10	24 ± 5
Breast cancer	40	72 ± 10	26 ± 4
Bladder cancer	40	72 ± 11	25 ± 4

Supplementary Table 2: The demographic characteristics of the cross-matched cohorts used in the multi-class classification analysis presented in Figure 3 (C).

Group	# Individuals	Age (years)	% Female	BMI (kg/m^2)
Stage I				
Cases	74	70 ± 9	45	26 ± 5
Controls	74	70 ± 9	45	26 ± 5
Stage II				
Cases	47	67 ± 10	43	26 ± 4
Controls	47	67 ± 10	43	27 ± 5
Stage III				
Cases	92	68 ± 10	43	26 ± 5
Controls	92	68 ± 10	43	27 ± 7
Stage IV				
Cases	142	68 ± 9	52	25 ± 5
Controls	142	68 ± 9	52	26 ± 4

Supplementary Table 3: Case-control design characteristics for the lung cancer stage analysis shown in Figure 5 of the main text.

Group	# Individuals	Age (years)	% Female	BMI (kg/m^2)
positive COPD lung cancer cases vs negative COPD controls				
COPD with Lung Cancer	116	68 ± 9	47	26 ± 5
Healthy	116	68 ± 9	47	27 ± 5
negative COPD lung cancer cases vs negative COPD controls				
Lung cancer without COPD	116	68 ± 10	50	25 ± 4
Healthy	116	68 ± 10	50	27 ± 5
positive COPD controls vs negative COPD controls				
COPD without Lung Cancer	323	68 ± 9	46	25 ± 6
Healthy	267	68 ± 9	42	26 ± 5
negative COPD LuCa cases vs positive COPD controls				
Lung Cancer without COPD	323	68 ± 10	48	25 ± 4
Controls with COPD	323	68 ± 9	47	25 ± 6

Supplementary Table 4: Case-control designs used in the analysis of the interplay of lung cancer with COPD presented in Figure 6 (A), (B), and (C) of the main text.

Group	# Individuals	Age (years)	% Female	BMI (kg/m^2)
positive diabetes LuCa cases vs non-symptomatic controls				
Lung Cancer with Diabetes	56	70 ± 9	36	28 ± 6
Healthy	56	70 ± 9	36	27 ± 4
negative diabetes LuCa cases vs non-symptomatic controls				
Lung Cancer without Diabetes	56	67 ± 10	48	25 ± 4
Healthy	56	67 ± 10	48	26 ± 5
positive kidney diseased LuCa cases vs non-symptomatic controls				
Lung Cancer with Kidney Disease	34	71 ± 7	38	27 ± 6
Healthy	34	71 ± 7	38	26 ± 6
negative kidney diseased LuCa cases vs non-symptomatic controls				
Lung Cancer without Kidney Disease	34	67 ± 10	50	26 ± 5
Healthy	34	67 ± 10	50	27 ± 5
Lung Cancer cases vs non-symptomatic controls among smokers				
Lung Cancer smokers	51	60 ± 6	55	25 ± 4
Healthy smokers	51	57 ± 10	63	26 ± 4
Lung Cancer cases vs NSR controls among non-smokers				
Lung Cancer non-smokers	49	68 ± 12	69	26 ± 4
Healthy non-smokers	49	68 ± 12	65	26 ± 5

Supplementary Table 5: Case-control designs used in the analysis of lung cancer comorbidities (kidney disease and diabetes) presented in Figure 6 (D) and (E) as well as smoking status shown in Figure 6 (F) of the main text.

Group	# Individuals	Age (years)	BMI (kg/m^2)
Female Patients			
Cases	218	67 ± 10	25 ± 5
Controls	218	62 ± 9	25 ± 5
Male Patients			
Cases	251	69 ± 9	26 ± 4
Controls	251	59 ± 14	27 ± 5

Supplementary Table 6: Characteristics of the age-matched case-control designs used in the analysis of different sex groups, shown in panel (A) of Supplementary Figure 2.

Group	# Individuals	Age (years)	% Female	BMI (kg/m^2)
First Age Group				
Cases	172	58 ± 5	52	26 ± 5
Controls	172	58 ± 5	52	26 ± 7
Second Age Group				
Cases	145	69 ± 3	47	26 ± 4
Controls	145	69 ± 5	54	26 ± 5
Third Age Group				
Cases	152	79 ± 3	40	25 ± 4
Controls	152	75 ± 5	45	26 ± 5

Supplementary Table 7: Characteristics of age- and sex-matched case-control designs used in the analysis of different age groups, shown in panel (B) of Supplementary Figure 2.

Group	# Individuals	Age (years)	% Female	BMI (kg/m^2)
		Underweight		
Cases	26	66 ± 10	77	18 ± 1
Controls	26	45 ± 17	88	18 ± 1
		Healthy Weight		
Cases	209	68 ± 10	53	22 ± 2
Controls	209	61 ± 10	66	22 ± 2
		Overweight		
Cases	232	69 ± 9	40	29 ± 3
Controls	232	63 ± 10	47	29 ± 5

Supplementary Table 8: Characteristics of age- and sex-matched case-control designs used in the analysis of different BMI groups, shown in panel (C) of Supplementary Figure 2.

Cancer type	EMF		FTIR		Dataset size (cases/controls)	
	Cross-validation	Test	Cross-validation	Test	Train set	Test set
LuCa	0.87 ± 0.03	0.80	0.90 ± 0.03	0.84	450/458	57/162
PrCa	0.69 ± 0.07	0.61	0.74 ± 0.07	0.66	284/285	132/127
BrCa	0.70 ± 0.09	0.57	0.76 ± 0.09	0.65	139/142	27/55
BlCa	0.67 ± 0.09	0.57	0.68 ± 0.07	0.59	170/177	31/182

Supplementary Table 9: ROC AUC values for binary classification of plasma fingerprints from cancer patients versus non-symptomatic controls, using cross-validation on training sets and independent testing on held-out test sets. Two measurement methods, EMF and FTIR, were applied. Note: The ROC AUC values for EMF-based models in this figure may differ from those in the main text due to variations in sample sizes, as these cohorts include only samples measured by both EMF and FTIR methods, for consistent comparability.

LuCa Stage	EMF		FTIR		Dataset size
	AUC	Mean effect size	AUC	Mean effect size	cases/controls
I	0.64 ± 0.15	0.36	0.63 ± 0.12	0.41	71/73
II	0.80 ± 0.15	0.69	0.79 ± 0.16	0.67	46/45
III	0.87 ± 0.07	0.66	0.87 ± 0.07	0.62	87/89

Supplementary Table 10: Stage-wise comparison of EMF- and FTIR-based model performance. The comparison is based on cross-validation ROC AUC values and average spectrally resolved effect sizes across wavenumbers that significantly contribute to class separation. Significant wavenumbers are identified via a t-test assessing differences between case and control mean values. Note: ROC AUC values for EMF-based models in this table may differ from those in the main text due to variations in sample sizes, as only samples measured by both EMF and FTIR methods are included for consistency.

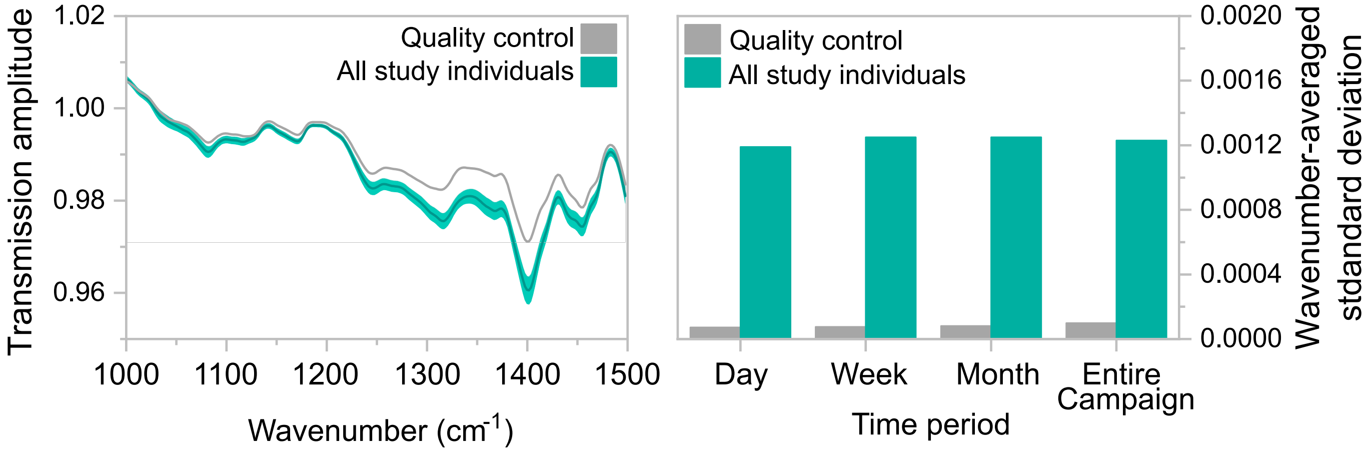

Supplementary Figure 3: FTIR fingerprinting reproducibility. (Left) Mean (solid line) and standard deviation of the transmission amplitude measured using a commercial FTIR spectrometer for quality control serum samples (grey) as well as blood plasma samples of individuals from the Lasers4Life study (cyan) measured over 5 months. (Right) The average value of the standard deviation across the spectral range from 950 cm^{-1} to 1375 cm^{-1} for the quality control (grey) and study samples (cyan) when considering measurements acquired over periods of a day, week, month, and the entire campaign. The wavenumber-averaged standard deviation of the quality control serum samples shown by the grey bars on the right panel roughly ranges from 0.3 % to 0.5 % of the biggest dip in the transmission amplitude shown by the grey solid line in the left panel, around 1315 cm^{-1}. This indicates an enhanced fingerprinting reproducibility for the FTIR instrument in comparison to the version of the EMF instrument described in this work. In the case of the study samples, as shown by the cyan bars, the value is around 4 %, which is comparable to the values shown by the cyan bars in the right panel of Fig. 1(c).
