# Supplementary material for: Electric-Field Molecular Fingerprinting to Probe Cancer
Source: ACS Cent Sci. 2025 Apr 9;11(4):560–73. doi: 10.1021/acscentsci.4c02164 (PMC12022918; doi:10.1021/acscentsci.4c02164)
Supplement: Supplementary file 2 — oc4c02164_si_002.pdf [file oc4c02164_si_002.pdf]

oc-2024-02164y.R1

Name: Peer Review Information for "Electric-field molecular fingerprinting to probe cancer"

First Round of Reviewer Comments

Reviewer: 1

Comments to the Author

The authors employ machine learning to classify the electric-field molecular fingerprint (EMF) of blood plasma in relation to cancer. Samples were collected from 2,533 patients as part of the Laser4Life clinical trial, where 1,392 patients diagnosed with cancer had not yet received treatment. The study evaluates the model's performance in detecting four cancer types and assesses its robustness against common co-morbidities that may influence molecular fingerprints. Notably, the authors provide statistically significant evidence for detecting lung cancer without relying on overly complex machine learning models. The use of EMFs, rather than Fourier-transformed infrared (FTIR) spectroscopy, is particularly advantageous due to its suitability for high-throughput applications and its immunity to signal interference from the excitation pulse. This study lays the groundwork for early cancer detection using minimally invasive techniques.

I recommend this paper for publication once the following points are addressed:

- Line 58: While the authors later clarify this, it is initially confusing that they emphasize the speed of a single EMF measurement (fractions of a second) and then mention a "40-second-long measurement." It would help to explain briefly how many measurements are taken within these 40 seconds and how they are processed (e.g., averaging) before being input into the machine learning algorithm.

- line 60: Similarly, it is unclear why high-throughput capabilities are highlighted, yet the measurements were conducted over seven months rather than a single week (assuming 2,500 samples x 4.5 minutes per sample). Clarifying this earlier would ease the reader's understanding.

- Fig 1: In the inset of part B, please add the delay times. Additionally, clarify what "250" represents—does it indicate signal amplification by a factor of 250 or 250 measurements?

- line 75: The phrase "solid black line" should be corrected to "solid grey line."

- line 100: Please reformulate the sentence "Case-control group pairs …" for clarity. Its current phrasing is difficult to follow.

- Fig 2: The insets in part B are unclear. The caption does not sufficiently explain them. What do the x- and y-axes represent? What standard deviation does the grey shaded region reflect, and why does it differ across the four insets? Since the pooled reference blood should exhibit consistent variation, this requires explanation. Additionally, clarify why these insets are relevant, given that the colored deviations are always smaller than one standard deviation. The deviations are notably larger for lung cancer (LuCa), but the significance of this difference needs to be explained. This clarification will also aid understanding of Figure 5A.

- line 138/139: Up to this point, it remains unclear why the authors performed 10-fold cross-validation (CV) rather than simply evaluating the hold-out set. This issue is related to the question raised regarding line 60. Concerning CV results, some AUC values are barely two standard deviations above 0.5. Were any of the 10 realizations below 0.5? If so, how many?

- Fig 5A: While this figure is clearer than the insets in Figure 2, the y-axis label is misleading, as it represents a difference rather than an EMF signal. Also, why does the x-axis cover only 500-1,200 fs instead of the 0-2,400 fs range shown in Figure 1?

- Line 198/199: The mathematical expression did not render correctly. More generally, what hypothesis test does the p-value relate to? It seems to be thrown in rather randomly.

- Fig 6: Is the third bar from the left labeled correctly? I would expect it to read "LuCa (COPD) vs positive COPD references."

- line 246: It is unclear what models were trained and for which ROC-AUC results are reported. Much of the text suggests the use of binary classifiers, yet the confusion matrices imply multi-class models. Are the ROC-AUC values only for the binary models?

- In general, how do the trained multi-class and binary models align with real-world applications? Would it make sense to first implement a binary model (cancer/healthy) followed by a multi-class model to identify the specific cancer type? How do the authors envision using their models for patient diagnosis?

- lines 262-265: The wording here implies that the three co-morbidities affected model robustness similarly, which is inconsistent with earlier claims that impaired kidney function had the most substantial impact.

- lines 340-341: Is it correct that the spectrometer collected 2,800x40 spectra and were these solely averaged to produce the waveforms shown in the text? For readers less familiar with EMF traces, could the authors explain briefly whether these signals have two axes (delay and frequency/wavenumber), akin to pump-probe spectroscopy? Also, please state precisely how the raw signals are transformed into the fingerprints used for machine learning. Are they simply the same?

- If the model input is truly simply the normalized EMF curve as a function of delay time, as shown in Fig 1, did the authors consider training the model directly on noisy traces, akin to adversarial training in image classification? Also, wouldn't a Fourier transform of this signal be more robust than the raw EMF curve?

- line 353: What is the dimensionality of the data? Understanding this would provide insight into why the authors opted for a "simple" logistic regression model rather than more complex architectures like fully connected networks or convolutional models (with a continuous filter) with a softmax layer. It seems that some signal processing or some ML architecture aimed at waves, e.g. sound, could have leveraged more out of the EMF data. This comment is based on my understanding that the model input were simply curves as those shown in Fig1B.

- line 354: You removed 46 spectra—out of how many? Does this correspond to 46 patient measurements or just 46 spectra out of the 2,000 x 2,533 collected?

- line 360: Why do the two listed scikit-learn versions differ so significantly?

Reviewer: 2

Comments to the Author

This manuscript presents statistically significant evidence from a proof-of-concept clinical study involving 2,533 participants, demonstrating that cancer can be detected by analyzing vibrational signatures in blood. This achievement represents an important step toward the long-standing goal of developing a non-invasive, cost-effective diagnostic test for clinical use. Techniques such as IR and Raman spectroscopy show great promise due to their sensitivity to molecular structures through vibrational analysis. In this study, the authors detect IR absorptions by analyzing their impact on femtosecond mid-IR pulses, as observed in the transmitted electromagnetic field. The findings highlight the critical importance of standardizing the approach, including spectroscopic measurements, data processing, and analysis, to ensure reliable and reproducible results.

Detecting stage I lung cancer is understandably challenging, as marker concentrations are minimal at this early stage. I concur with the conclusion's emphasis on future enhancements, particularly the need for broader spectral coverage, which is readily achievable with Raman spectroscopy. Have the authors considered detecting these vibrational signatures using time-resolved coherent anti-Stokes Raman scattering? Additionally, could interferometric subtraction be explored? If an "average" signal can be established for healthy individuals, subtraction might enhance the visibility of disease biomarkers.

I commend the authors for pursuing the ambitious goal of a non-invasive, cost-effective diagnostic test for clinical use. However, the current mid-IR source appears costly. The mention of quantum cascade lasers as an alternative is promising, and I believe resonance Raman spectroscopy could offer comparable benefits.

Overall, the manuscript is well-written and illustrated. I appreciate the authors' cautious approach, avoiding overstated claims while presenting their findings effectively.

Author's Response to Peer Review Comments:

Dear Editorial office, dear Reviewers,

We appreciate the Reviewers' constructive feedback and their recommendations. Below, we provide point-by-point responses to Reviewers' comments and formatting suggestions (in blue). Please find also enclosed a revised manuscript incorporating all changes (highlighted in red).

Reviewer(s)' Comments to Author:

Reviewer: 1

Recommendation: Publish in ACS Central Science after minor revisions noted.

Comments:

The authors employ machine learning to classify the electric-field molecular fingerprint (EMF) of blood plasma in relation to cancer. Samples were collected from 2,533 patients as part of the Laser4Life clinical trial, where 1,392 patients diagnosed with cancer had not yet received treatment. The study evaluates the model's performance in detecting four cancer types and assesses its robustness against common co-morbidities that may influence molecular fingerprints. Notably, the authors provide statistically significant evidence for detecting lung cancer without relying on overly complex machine learning models. The use of EMFs, rather than Fourier-transformed infrared (FTIR) spectroscopy, is particularly advantageous due to its suitability for high-throughput applications and its immunity to signal interference from the excitation pulse. This study lays the groundwork for early cancer detection using minimally invasive techniques.

We appreciate the Reviewers' careful assessment and estimation of the impact of our work.

I recommend this paper for publication once the following points are addressed:

- **Line 58: While the authors later clarify this, it is initially confusing that they emphasize the speed of a single EMF measurement (fractions of a second) and then mention a "40-second-long measurement." It would help to explain briefly how many measurements are taken within these 40 seconds and how they are processed (e.g., averaging) before being input into the machine learning algorithm.**

We acknowledge the lack of clarity regarding the acquisition time and have thus added the following sentence at line 59:

"Each 40-second-long EMF signal measurement was obtained by averaging 112,000 individual traces."

- **line 60: Similarly, it is unclear why high-throughput capabilities are highlighted, yet the measurements were conducted over seven months rather than a single week (assuming 2,500 samples x 4.5 minutes per sample). Clarifying this earlier would ease the reader's understanding.**

We agree with the Reviewer and have added the following sentence at line 63:

“The daily time allotted for the clinical study samples was limited due to the two-hour stabilization period required by the laser source and the time needed to measure samples not included in this study.”

- **Fig 1: In the inset of part B, please add the delay times. Additionally, clarify what "250" represents—does it indicate signal amplification by a factor of 250 or 250 measurements?**

We appreciate the Reviewer’s careful assessment here. For clarity, we have made the following changes. The sentence at line 67 has been revised as follows:

“The inset displays a magnified view of the EMF signals in the delay range from 1050 to 1150 femtoseconds.”

Moreover, we have further added the delay labels to the inset in Figure 1(B) and included the following sentence in the figure caption:

“The inset zooms into the EMF signals in the delay range from 1050 to 1150 femtoseconds, with the signal values amplified by a factor of 400 along the y-axis.”

- **line 75: The phrase "solid black line" should be corrected to "solid grey line."**

The necessary correction has been implemented, we thank the Reviewer for highlighting it.

- **line 100: Please reformulate the sentence "Case-control group pairs ..." for clarity. Its current phrasing is difficult to follow.**

For better understanding, the sentence beginning at line 102 has been revised as follows:

“The capacity of EMF to aid cancer diagnostics was tested in the multi-centric Lasers4Life clinical study conducted in the Munich area, where the study participants were divided into case-control group pairs of therapy-naïve cancer patients (with cancer of either the lung, prostate, breast or bladder) and asymptomatic control individuals.”

- **Fig 2: The insets in part B are unclear. The caption does not sufficiently explain them. What do the x- and y-axes represent? What standard deviation does the grey shaded region reflect, and why does it differ across the four insets? Since the pooled reference blood should exhibit consistent variation, this requires explanation. Additionally, clarify why these insets are relevant, given that the colored deviations are always smaller than one standard deviation. The deviations are notably larger for**

lung cancer (LuCa), but the significance of this difference needs to be explained. This clarification will also aid understanding of Figure 5A.

We agree with the Reviewer and have revised the caption to provide a clearer explanation of the insets:

"Insets display the mean difference in EMF signals between cancer patients and control individuals (solid line), along with the standard deviation in the EMF signal of the corresponding controls (grey-shaded region). The x-axis represents the delay, ranging from 500 to 1200 femtoseconds. The y-axis scale is identical across all four insets, ensuring direct comparability."

As noted above, the shaded areas represent the standard deviation of the EMF signal for the spectroscopic measurements of control groups used in each matched cohort. Because the sampled individuals - and thus also their respective spectroscopic measurements - differ for each of the cancer entities due to variations in demographic and anthropometric characteristics, the variability between different control groups naturally differs as well. For example, the measured spectral variability in lung cancer control individuals differs from the variability of breast cancer control individuals.

Comparing the mean of the measured spectral differences to the standard deviation of the control individuals provides a measure of effect size over time. While this metric does not add significant information beyond the ROC curves, it enhances interpretability by offering a visual representation of the results. For instance, it illustrates why lung cancer is more easily detectable than other cancer types, reinforcing the results provided by the ROC analysis.

- line 138/139: Up to this point, it remains unclear why the authors performed 10-fold cross-validation (CV) rather than simply evaluating the hold-out set. This issue is related to the question raised regarding line 60. Concerning CV results, some AUC values are barely two standard deviations above 0.5. Were any of the 10 realizations below 0.5? If so, how many?

We performed cross-validation (CV) rather than evaluating a single hold-out set to obtain a more robust and reliable estimate of our model's performance on data drawn from the same distribution. A hold-out set evaluation alone may yield a biased or high-variance estimate, particularly when the dataset is small. In contrast, CV leverages multiple training-testing splits to produce a more stable and generalizable assessment of the model's performance. The held-out test set serves a different purpose: to evaluate the model's performance under non-identical conditions.

We appreciate the Reviewer's request for clarification regarding the CV results. The performance evaluation method on the training set is now described in the Methods section in the revised manuscript as follows (line 369):

"Performance evaluation on the training data set was conducted using a nested cross-validation approach. Hyperparameter optimization was performed through a 5-fold grid search cross-validation nested within a repeated stratified 10-fold cross-validation with five repetitions. The results are visualized through ROC curves."

Additionally, within the nested cross-validation framework, 50 AUC realizations were generated for each cancer entity. Below is the count of realizations with an AUC below 0.5:

- LuCa vs NSR: 0 out of 50
- PrCa vs NSR: 0 out of 50
- BrCa vs NSR: 1 out of 50
- BlCa vs NSR: 0 out of 50

These results confirm that the main portion of the AUC value distributions is well above chance levels.

- **Fig 5A: While this figure is clearer than the insets in Figure 2, the y-axis label is misleading, as it represents a difference rather than an EMF signal. Also, why does the x-axis cover only 500-1,200 fs instead of the 0-2,400 fs range shown in Figure 1?**

As suggested, we have corrected the y-axis label in Figure 5(A). The figure displays a limited x-axis range for better visualization. The caption for Figure 5(A) has been revised as follows:

"The mean difference in measured plasma EMF signals between cancer patients and control individuals (solid line) and the standard deviation in the EMF signal for the control individuals (grey-shaded region), plotted against the time delay ranging from 500 to 1200 femtoseconds for better visibility, stratified by lung cancer stage."

- **Line 198/199: The mathematical expression did not render correctly. More generally, what hypothesis test does the p-value relate to? It seems to be thrown in rather randomly.**

To avoid confusion, we have removed the p-value from the main text. This passage refers to results presented in Supplementary Table 10, whose caption clarifies the statistical reasoning behind identifying wavenumbers that significantly contribute to class separation. As stated, significant wavenumbers are identified via a t-test assessing differences between case and control mean values.

- **Fig 6: Is the third bar from the left labeled correctly? I would expect it to read "LuCa (COPD) vs positive COPD references."**

Yes, the bar is correctly labeled. This result was obtained by analyzing whether EMF captures COPDspecific signals. To this end, we constructed a case-control design to compare COPD patients with nonsymptomatic reference subjects.

The case 'LuCa (COPD) vs. positive COPD references' was not explored.

- **line 246: It is unclear what models were trained and for which ROC-AUC results are reported. Much of the text suggests the use of binary classifiers, yet the confusion matrices imply multi-class models. Are the ROC-AUC values only for the binary models?**

The ROC-AUC values within the manuscript are provided only for the binary classification models. Multiclass classification models are used solely for the evaluation presented in Fig. 3(c).

- **In general, how do the trained multi-class and binary models align with real-world applications? Would it make sense to first implement a binary model (cancer/healthy) followed by a multi-class model to identify the specific cancer type? How do the authors envision using their models for patient diagnosis?**

We envision binary classification models being applicable to aid primary diagnostic purposes for specific groups of individuals. In our study, individuals were enrolled and venous blood sampled before primary diagnosis. The study aimed to examine whether EMF can serve as a minimally invasive paradigm before any therapeutic interventions.
Thus, the multi-class classification analysis is conducted solely for purposes to determine whether the EMF signals we capture are disease-specific or reflect a more general pattern of molecular deviation (e.g., inflammation). The demonstrated ability of EMF-based multi-class classifiers to distinguish between different cancer types provides evidence that cancer-specific signals are encoded in EMF signals.

- **lines 262-265: The wording here implies that the three co-morbidities affected model robustness similarly, which is inconsistent with earlier claims that impaired kidney function had the most substantial impact.**

We wish to note that in the listed passage, we aimed to indicate that the three co-morbidities influence model robustness to varying degrees, but we do not suggest that their effects are identical. The key finding is that the investigated co-morbidities do not prevent cancer detection with our approach.

Given the suggestion of the Reviewer, we incorporated the following statement, line 266:
"In the context of lung cancer detection, while abnormal kidney function impacted model accuracy, the models effectively distinguished lung cancer patients from matched individuals despite common comorbidities such as COPD and type-2 diabetes mellitus."

- **lines 340-341: Is it correct that the spectrometer collected 2,800x40 spectra and were these solely averaged to produce the waveforms shown in the text? For readers less familiar with EMF traces, could the authors explain briefly whether these signals have two axes (delay and frequency/wavenumber), akin to pump-probe spectroscopy? Also, please state precisely how the raw signals are transformed into the fingerprints used for machine learning. Are they simply the same?**

We would like to thank the Reviewer for pointing out the lack of clarity in this section. Indeed, the 2,800 x 40 spectra, after applying the pre-processing steps, including interpolation to a common delay axis, were averaged to produce the waveforms shown in the manuscript, with the EMF signal as a function of delay. At each delay point, the instrument captures a single value representing the electric field corresponding to the delay and not a spectral distribution as in pump-probe spectroscopy. An EMF trace thus captures spectroscopic information in the time domain. Fourier transformation of such a trace gives the corresponding spectral information, as it could be measured with a dispersing spectrometer, as it is typically used in visible to near-infrared range, also for pump-probe measurements. The individual or averaged traces carry the stationary spectroscopic information of the sample. To be clearer, we do not have a second axis here with additional time delay as in pump-probe spectroscopy. Also, as we measure the electric field and not the time-integrated intensities, in addition to the intensity spectrum, we obtain the spectral phase information by Fourier transformation. The averaged EMF traces undergo a standardization process, including applying the Fourier transform to obtain the complex spectra, division of each sample spectrum by its corresponding reference water spectrum, and time-domain filtering. The steps involved in this standardization process, aimed at suppressing the noise of the ultrashort excitation process, are described in detail in Ref. 39 (Marinus Huber et al., "Standardized Electric-Field-Resolved Molecular Fingerprinting", Analytical Chemistry 2024 96 (32), 13110-13119).

Along with the Reviewer's suggestions, we have made the following changes in the Methods section, under the sub-section, "Pre-processing and standardization of electric-field molecular fingerprinting measurements", to enhance clarity. The sentence starting at line 346 has been modified as follows:

"The EMF signals, acquired at a rate of 2800 traces per second, were calibrated, interpolated to a common delay axis, and averaged to obtain a single trace with the EMF signal as a function of delay in femtoseconds for each 40-second-long measurement, similar to the traces shown in Figure 1(B)."

Furthermore, an additional sentence has been included (line 355):

"The standardized fingerprints constitute the input data sets for subsequent machine-learning-based classification analyses."

- **If the model input is truly simply the normalized EMF curve as a function of delay time, as shown in Fig 1, did the authors consider training the model directly on noisy traces, akin to adversarial training in image classification? Also, wouldn't a Fourier transform of this signal be more robust than the raw EMF curve?**

We thank the Reviewer for the thoughtful considerations. The model input consists of standardized EMF time traces. The standardization process—comprising Fourier transformation, time-domain filtering, and inverse Fourier transformation—enhances signal robustness compared to raw EMF signals. Using noisy traces directly offers no advantage; our experiments showed that models trained on noisy time traces performed worse on the held-out test dataset.

After standardization, we chose to analyze EMF directly in the time domain. Classification models trained in both the time and Fourier domains yielded nearly identical results, which is expected since both representations contain the same information.

- **line 353: What is the dimensionality of the data? Understanding this would provide insight into why the authors opted for a "simple" logistic regression model rather than more complex architectures like fully connected networks or convolutional models (with a continuous filter) with a softmax layer. It seems that some signal processing or some ML architecture aimed at waves, e.g. sound, could have leveraged more out of the EMF data. This comment is based on my understanding that the model input were simply curves as those shown in Fig1B.**

Each dataset consists of 20,000 features, capturing the EMF signal at various time points. The high dimensionality, coupled with strong correlations among temporal features, suggests that linear models like logistic regression are well-suited for analysis. While more complex deep learning approaches, such as convolutional neural networks (CNNs), have been explored, they have not demonstrated a clear advantage. We are continuing to investigate their potential benefits, in a separate project.

- **line 354: You removed 46 spectra—out of how many? Does this correspond to 46 patient measurements or just 46 spectra out of the 2,000 x 2,533 collected?**

We applied an outlier detection method, which resulted in the removal of 46 outlier spectra, corresponding to 46 individuals. These individuals were excluded before defining the matched cohorts used in the study. This procedure is detailed in the Methods section, subsection “Outlier Detection”. For clarity, we have rewritten the last sentence of this subsection as follows (lines 361):

“This procedure led to the removal of 46 spectra, which were excluded before defining the matched cohorts used in the study.”

- **line 360: Why do the two listed scikit-learn versions differ so significantly?**

We thank the Reviewer for highlighting this inconsistency. It was a typo, and we have corrected it.

Reviewer: 2

Recommendation: Publish in ACS Central Science without change.

Comments:

This manuscript presents statistically significant evidence from a proof-of-concept clinical study involving 2,533 participants, demonstrating that cancer can be detected by analyzing vibrational signatures in blood. This achievement represents an important step toward the long-standing goal of developing a non-invasive, cost-effective diagnostic test for clinical use. Techniques such as IR and Raman spectroscopy show great promise due to their sensitivity to molecular structures through vibrational analysis. In this study, the authors detect IR absorptions by analyzing their impact on femtosecond mid-IR pulses, as observed in the transmitted electromagnetic field. The findings highlight the critical importance of standardizing the approach, including spectroscopic measurements, data processing, and analysis, to ensure reliable and reproducible results.

Detecting stage I lung cancer is understandably challenging, as marker concentrations are minimal at this early stage. I concur with the conclusion's emphasis on future enhancements, particularly the need for broader spectral coverage, which is readily achievable with Raman spectroscopy. Have the authors considered detecting these vibrational signatures using time-resolved coherent antiStokes Raman scattering? Additionally, could interferometric subtraction be explored? If an "average" signal can be established for healthy individuals, subtraction might enhance the visibility of disease biomarkers.

The Reviewer is correct in noting that Raman spectroscopy could provide broader spectral coverage more easily. However, this would come at the cost of significantly reduced sensitivity. Our preference is to eventually achieve both. Towards this end are actively working on expanding the spectral coverage to more than 2 octaves (up from the current less than 1 octave), as outlined in reference 40 (P. Steinleitner, N. Nagl, M. Kowalczyk et al., "*Single-cycle Infrared Waveform Control*," Nature Photonics 16, 512-518 (2022)), which forms the foundation for next-generation EMF. Interferometric subtraction is an excellent suggestion that we are also already exploring. Towards this end, we have added the following sentence to the discussion (line 276):

"Interferometric subtraction of EMF signals could enhance detection sensitivity by suppressing the technical noise arising from the impulsive excitation pulse (T. Buberl et al., "Broadband interferometric subtraction of optical fields", Optics Express 27, 2432-2443 (2019))."

I commend the authors for pursuing the ambitious goal of a non-invasive, cost-effective diagnostic test for clinical use. However, the current mid-IR source appears costly. The mention of quantum cascade lasers as an alternative is promising, and I believe resonance Raman spectroscopy could offer comparable benefits.

Overall, the manuscript is well-written and illustrated. I appreciate the authors' cautious approach, avoiding overstated claims while presenting their findings effectively.

We wish to thank the Reviewer for their encouraging feedback.

Formatting needs:

AFFILIATIONS: Please ensure that the full details of author affiliations are listed in the manuscript file. The following details are required: institution, city, postal code (if existing), and country.

The correctness of the affiliations has been verified. Additionally, we have now included the postal code for each institution.

SYNOPSIS MISSING: ACS Central Science requires a brief synopsis. The synopsis should be no more than 200 characters (including spaces) and should reasonably correlate with the Table of Contents (TOC) graphic. The synopsis is intended to explain the importance of the article to a broader readership across the sciences. Please place your synopsis in the manuscript file after the TOC graphic.

We would like to include the following synopsis:

"Laser-based infrared molecular fingerprinting detects cancer, demonstrating its potential for clinical disease diagnostics."

SI PARAGRAPH MISSING: Please provide a brief description of the contents of the supplementary material at the end of the manuscript file before the Acknowledgments and References sections with the heading "Supporting Information." Please avoid long paragraphs or lists of Supplemental figure captions. Examples of sufficient and insufficient descriptions are below:

-SUFFICIENT:

"1H NMR spectra for all compounds" or

"Additional experimental details, materials, and methods, including photographs of experimental setup".

-INSUFFICIENT:

"Figures S1-S3" or

"Additional figures as mentioned in the text".

We have included the following description of the supplementary materials at the end of the manuscript, just before the Acknowledgments section:

"Additional details on the study cohort characteristics and supplementary figures are provided in a separate file accompanying this manuscript to support the main text."

SI GRAPHICS: Please remove Supporting Information figures/tables/legends from your manuscript and upload as a separate file with title page. Save under "Supporting Information for Publication" file designation.

The supporting information has been removed from the main manuscript and is now provided as a separate document.

SI HEADER: Please ensure that the Supporting Information file has a title page with a "Supporting Information" heading, manuscript title and author names, exactly as they appear on the title page of the manuscript.

The supporting information document is titled with a "Supporting Information" heading, followed by the manuscript title and author names.

oc-2024-02164y.R2

Name: Peer Review Information for "Electric-field molecular fingerprinting to probe cancer"

Second Round of Reviewer Comments

Author's Response to Peer Review Comments:

Dear Editor,

It is my great pleasure to learn that our paper has been provisionally accepted for publication in ACS Central Science. On behalf of all my coauthors, I would wish to thank you for very smooth manuscript processing and securing reviewers with constructive feedback.

Please find all requested manuscript files with minor edits uploaded.

Sincerely,

Mihaela Zigman.

Dr. Mihaela Žigman

Max Planck Institute of Quantum Optics

Laboratory for Attosecond Physics

Hans-Kopfermann-Str. 1, 85748 Garching, Germany

Ludwig-Maximilians-Universität München

Chair of Experimental Physics — Laser Physics

Centre for Advanced Laser Applications

Am Coulombwall 1, 85748 Garching, Germany
